# Enhanced External Counterpulsation Versus Standard Medical Therapy for Chronic Refractory Angina: A Systematic Review and Meta-Analysis

**DOI:** 10.3390/biomedicines14092093

**Published:** 2026-09-17

**Authors:** Praneeth Ulavala, Maruf Sarwar, Utkrash Mishra, Stephen Deji Adedokun, Gilbert-Roy Kamoga

**Affiliations:** 1 Department of Internal Medicine, College of Medicine-Northwest, University of Arkansas Medical Science, Fayetteville, AR 72703, USA; pulavala@uams.edu (P.U.); gkamoga@uams.edu (G.-R.K.); 2 Division of Cardiovascular Medicine, College of Medicine, University of Tennessee Health Science Center, Memphis, TN 38103, USA; mutkarsh@uthsc.edu; 3 University of Tennessee Health Science Library, Memphis, TN 38103, USA; 4 Division of Cardiovascular Medicine, Weil Cornell Medical College, New York, NY 10065, USA; uqn9004@med.cornell.edu

**Keywords:** enhanced external counterpulsation, coronary artery disease, angina, myocardial infarction, INOCA, MINOCA

## Abstract

**Background:** Chronic refractory angina is a disabling syndrome of persistent ischemic symptoms despite guideline-directed medical therapy and, in many patients, prior coronary revascularization. Enhanced external counterpulsation [EECP] is a noninvasive therapy that augments diastolic coronary perfusion, reduces systolic afterload, improves endothelial function, and may promote collateral recruitment. Although developed for obstructive coronary artery disease, its vascular and microvascular effects may also be relevant to contemporary ischemic syndromes, including ischemia with non-obstructive coronary arteries [INOCA] and myocardial infarction with non-obstructive coronary arteries [MINOCA]. A contemporary quantitative synthesis of EECP across angina burden, functional capacity, quality of life, and safety is therefore warranted. **Methods:** We performed a systematic review and meta-analysis of randomized trials and prospective studies of adults with chronic refractory angina treated with EECP versus standard medical therapy, usual care, or sham EECP. Outcomes were Canadian Cardiovascular Society [CCS] angina class, nitroglycerin use, the 6 min walk test [6MWT], treadmill exercise testing, quality-of-life measures, adverse events, and treatment withdrawal. Because the eligible evidence combined sham-controlled randomized trials with single-arm and registry cohorts, pooled estimates were derived using random-effects models and are interpreted in that context. **Results:** Twenty-six studies were included. EECP was associated with improvement across every prespecified domain: CCS angina class [standardized mean difference [SMD] −1.56; 95% confidence interval [CI], −2.54 to −0.58; *p* = 0.002], nitroglycerin use [SMD −2.41; 95% CI, −3.34 to −1.49; *p* < 0.00001], 6MWT [SMD 1.59; 95% CI, 0.64 to 2.54; *p* = 0.001], treadmill exercise testing [SMD 1.03; 95% CI, 0.15 to 1.91; *p* = 0.02], and quality of life [SMD 1.12; 95% CI, 0.71 to 1.53; *p* < 0.00001]. Heterogeneity was high for all pooled outcomes [I^2^ ≥ 79%], and the largest effect sizes were driven substantially by uncontrolled before–after data. **Conclusions:** EECP is associated with statistically significant and clinically meaningful reductions in angina burden and nitrate use, with parallel improvements in objective exercise capacity and patient-reported quality of life among patients with chronic refractory angina. These findings support EECP as a reasonable adjunctive option for appropriately selected patients who remain symptomatic despite conventional treatment, while the magnitude of heterogeneity and the contribution of non-randomized data temper the certainty of the pooled estimates and underscore the need for standardized protocols, contemporary sham-controlled trials, and dedicated investigation in INOCA and MINOCA populations.

## 1. Introduction

Chronic refractory angina refers to persistent angina pectoris caused by myocardial ischemia in patients with coronary artery disease who remain symptomatic despite guideline-directed medical therapy and who are not amenable to further revascularization with percutaneous coronary intervention (PCI) or coronary artery bypass grafting (CABG) [1]. As survival after acute coronary syndromes has improved and the population ages, clinicians increasingly encounter highly symptomatic, frequently revascularized “no-option” patients, a growing group that experiences substantial impairment in functional capacity, recurrent health-care utilization, and diminished quality of life despite apparently optimized antianginal regimens [2]. Contemporary reviews emphasize that durable, effective options for these patients remain scarce, motivating renewed interest in noninvasive adjuncts [3].

Enhanced external counterpulsation (EECP) is a noninvasive treatment in which pneumatic cuffs applied to the calves, thighs, and buttocks inflate sequentially during diastole and deflate immediately before systole. This timing increases venous return and diastolic coronary perfusion while reducing systolic afterload. Beyond these immediate hemodynamic effects, repeated EECP sessions appear to increase beneficial pulsatile endothelial shear stress, improve nitric oxide bioavailability, reduce arterial stiffness, and promote collateral vessel recruitment. On the strength of early trials, EECP entered clinical practice as an option for refractory angina, albeit with a modest recommendation grade reflecting the limited randomized evidence available at the time [4]. Trial-level estimates of benefit have since varied because of differences in study design, comparator arms, treatment intensity, baseline disease severity, and follow-up duration.

The objective of this systematic review and meta-analysis was to quantify the effect of EECP on angina symptoms, nitrate utilization, functional capacity, quality of life, and safety in patients with chronic refractory angina and to situate these effects within a contemporary understanding of ischemic syndromes that increasingly include non-obstructive coronary disease [3].

## 2. Study Population and Clinical Context

Across the included studies, patients generally had refractory angina despite optimized pharmacologic therapy and previous coronary intervention. Definitions of refractory angina varied, although most studies enrolled patients with CCS class III to IV symptoms who were considered unsuitable for additional revascularization [5]. Mean age generally ranged from 59 to 69 years, and men represented the majority of participants, accounting for approximately 65% to 86% of study cohorts [6,7,8].

The burden of coronary disease was substantial. Several cohorts reported three-vessel coronary artery disease in approximately 70% to 73% of patients, and prior PCI or CABG was common, with some studies reporting previous revascularization in 93% to 100% of participants [9]. Left ventricular systolic function was often preserved or mildly reduced, with reported ejection fractions of approximately 48% to 52%, although selected studies deliberately included patients with ischemic heart failure or reduced ejection fraction [10,11].

Traditional cardiovascular risk factors were highly prevalent. Diabetes mellitus, hypertension, and hyperlipidemia were frequently reported comorbidities [6]. Several studies specifically examined diabetes as a potential modifier of treatment response, reflecting the biologic plausibility that microvascular dysfunction and endothelial impairment may influence the magnitude of benefit derived from EECP [12,13,14].

## 3. Methods

**Figure 1:**
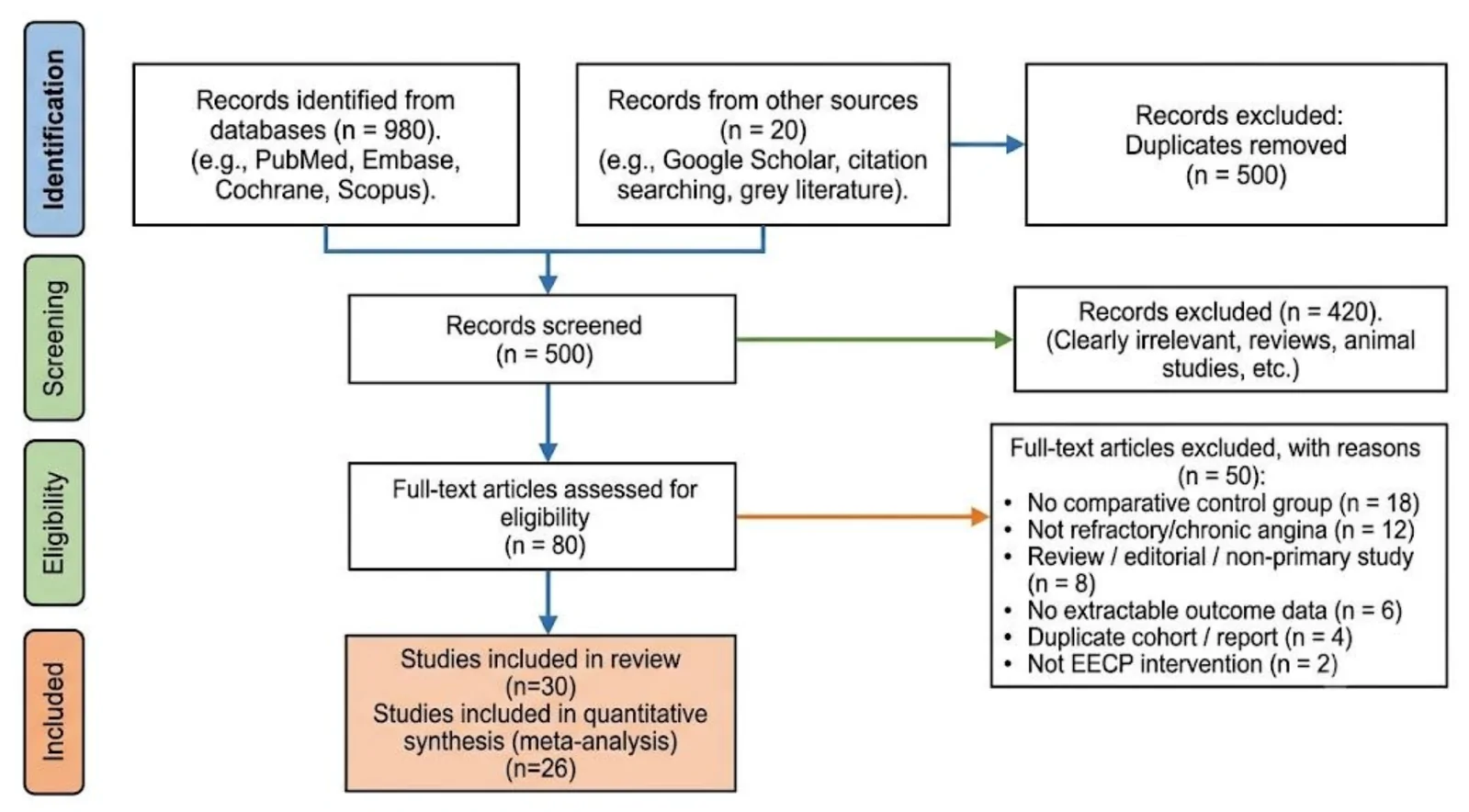
PRISMA flowchart.

This review was conducted in accordance with PRISMA principles, as outlined in Figure 1. A systematic search strategy was developed with medical librarian input and applied to PubMed/MEDLINE, Embase, and Cochrane CENTRAL. The search combined controlled vocabulary and free-text terms for enhanced external counterpulsation and refractory or chronic angina. After removal of duplicate records, two reviewers independently screened titles, abstracts, and full-text articles against prespecified eligibility criteria, and disagreements were resolved by consensus.

### 3.1. Eligibility Criteria and PICO Framework

Population: Adults [≥18 years] with chronic refractory angina attributable to coronary artery disease, persistent symptoms despite medical therapy, or ineligibility for further revascularization.

Intervention: EECP therapy, most commonly delivered as a conventional 35-session course consisting of 1 h daily treatments.

Comparator: Standard medical therapy, usual care, conservative therapy, or sham EECP.

Outcomes: Angina severity, including CCS class and nitroglycerin use; exercise capacity, including 6MWT and treadmill parameters; quality-of-life outcomes; and safety, including adverse events and treatment withdrawal.

### 3.2. Search Strategy

The following core search string was used and adapted as appropriate for each database: [“Angina Pectoris”[Mesh] OR “Microvascular Angina”[Mesh] OR “refractory angina”[tiab] OR “intractable angina”[tiab] OR “chronic stable angina”[tiab] OR “chronic angina”[tiab] OR “no-option angina”[tiab] OR “angina pectoris”[tiab]] AND [“Counterpulsation”[Mesh] OR “enhanced external counterpulsation”[tiab] OR “external counterpulsation”[tiab] OR “EECP”[tiab] OR “ECP”[tiab]]. Reference lists of relevant reviews and included studies were reviewed to identify additional eligible studies.

### 3.3. Data Extraction and Statistical Analysis

Study characteristics, patient demographics, EECP protocol details, comparator type, follow-up duration, and outcome data were extracted in duplicate when available. Continuous outcomes were pooled using standardized mean differences (SMDs) to accommodate variation in measurement scales across studies. Random-effects models were used because of expected clinical and methodologic heterogeneity, including differences between sham-controlled randomized trials and prospective observational cohorts [13]. Because eligible studies spanned sham-controlled randomized trials, prospective single-arm cohorts, and registry analyses, this design heterogeneity was anticipated a priori and is reflected both in the choice of random-effects modeling and in the cautious, design-aware interpretation applied throughout.

Effect measures are presented as SMDs with 95% confidence intervals (CIs). Negative SMDs favor EECP for outcomes in which lower values indicate improvement, such as CCS class or nitroglycerin use; positive SMDs favor EECP for outcomes in which higher values indicate improvement, such as 6MWT distance, exercise duration, and quality-of-life scores. Between-study heterogeneity was assessed using the I^2^ statistic, with values >50% considered substantial [14]. Given the consistently high heterogeneity observed across outcomes, pooled estimates were interpreted in the context of study design, comparator selection, baseline severity, and follow-up interval rather than as a single uniform treatment effect. Importantly, randomized sham-controlled trials and non-randomized studies were not considered to provide equivalent levels of causal evidence. Uncontrolled before–after and observational studies are particularly susceptible to regression to the mean, placebo effects, temporal trends, confounding, and co-interventions, which may exaggerate the apparent magnitude of treatment effects. Accordingly, pooled estimates incorporating non-randomized evidence were interpreted as estimates of the overall observed effect across heterogeneous study designs rather than as definitive causal estimates of EECP efficacy. Particular caution was applied to outcomes in which large effects were driven predominantly by uncontrolled or observational studies, and such findings were considered hypothesis-generating.

### 3.4. Appraisal of Study Quality

A prespecified sensitivity analysis was planned to evaluate the influence of non-randomized studies on the overall findings by restricting the analysis to randomized sham-controlled trials, thereby assessing whether observed effects remained when the evidence was limited to studies with the greatest protection against confounding and placebo effects. For outcomes reported by two or more randomized sham-controlled trials, pooled effect estimates were to be recalculated using the same random-effects model as the primary analysis. When only a single randomized trial reported an outcome, results were summarized descriptively without quantitative pooling. This design-stratified approach was intended to determine whether the observed treatment effects persisted when analysis was limited to the highest-quality evidence, thereby minimizing the potential influence of placebo effects, regression to the mean, and co-interventions that cannot be adequately controlled in single-arm before–after or observational studies.

Study design was therefore explicitly considered during interpretation of the pooled estimates. Randomized sham-controlled trials were regarded as providing the strongest evidence for a causal treatment effect, whereas prospective single-arm and observational studies were considered more vulnerable to systematic differences between baseline and follow-up measurements. Consequently, substantial effects observed in the overall analysis were not assumed to represent causal treatment effects unless supported by the randomized sham-controlled evidence. Where the direction or magnitude of effects differed between the overall analysis and the randomized-only sensitivity analysis, greater weight was given to the randomized evidence.

## 4. Results

### 4.1. Study Characteristics

The review included randomized, sham-controlled trials as well as prospective observational studies and registry-based cohorts. This variation in study design resulted in important differences in the strength of causal inference across the included evidence, with randomized sham-controlled trials providing the most robust assessment of treatment efficacy and non-randomized studies primarily contributing information regarding changes observed following EECP exposure. Participants were predominantly older men with advanced coronary artery disease, a high prevalence of prior PCI or CABG, and persistent angina despite conventional therapy. Comparators varied across studies and included sham EECP, usual care, and standard medical therapy. Most studies evaluated the conventional EECP regimen of approximately 35 h, although several contemporary cohorts examined alternative or repeated treatment schedules. Table 1 summarizes the representative studies and their principal contributions, and Table 2 presents the pooled quantitative estimates for each prespecified outcome.

### 4.2. Effects on Angina Severity

#### 4.2.1. Canadian Cardiovascular Society Classification

Eight studies, including cohorts reported by Shechter, Braverman, Lawson, Barsheshet, and others, contributed 1860 participants to the pooled analysis of CCS angina class [5,9,12,16,17,18,27,28]. EECP was associated with a significant reduction in angina severity compared with control therapy [SMD −1.56; 95% CI, −2.54 to −0.58; *p* = 0.002], although heterogeneity was substantial [I^2^ = 96%]. The direction of effect was consistently favorable, and the magnitude of improvement generally corresponded to at least a 1-class reduction in CCS functional status.

Individual studies support the pooled direction of effect. In the sham-controlled trial by Braith et al., 62% of patients receiving active EECP improved in angina class, whereas no improvement was observed in the sham-control group [*p* < 0.001] [16]. Shechter et al. reported improvement in mean CCS class from 3.5 ± 0.5 to 1.9 ± 0.3 [*p* < 0.0001], and Barsheshet et al. observed median improvement from class 3.0 to class 2.0 [*p* < 0.001] [5,9].

**Figure 2:**
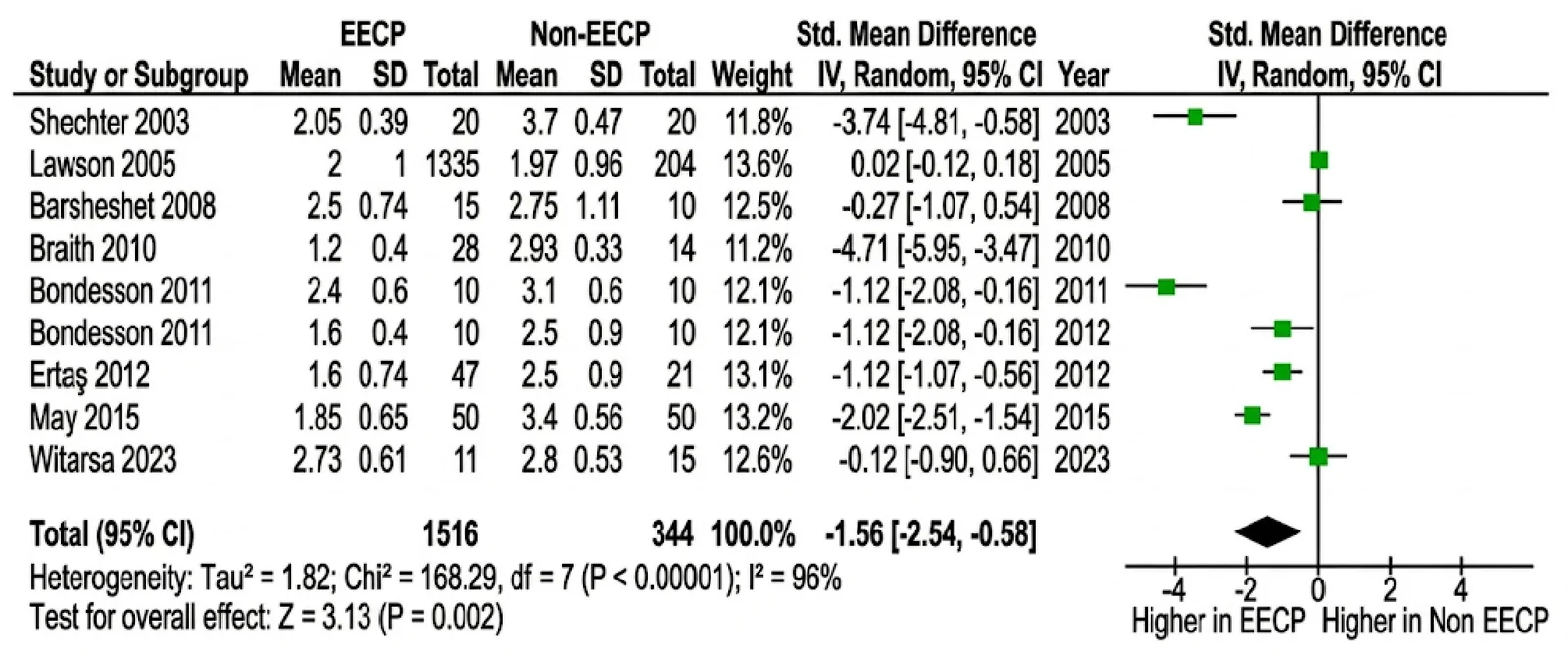
Forest plot of the effect of enhanced external counterpulsation [EECP] on Canadian Cardiovascular Society [CCS] angina class compared with control therapy [5,9,12,16,17,18,27,28].

Durability of angina relief was also reported in longer-term cohorts. May et al. found that 76% of patients maintained at least a 1-class improvement in CCS status at 12 months [17], as seen in Figure 2. In the International EECP Patient Registry, Lawson et al. reported persistence of clinical benefit at 2 years, with only 16% to 23% of patients remaining in CCS class III or IV compared with 83% to 92% at baseline [18]. However, Erdling et al. reported attenuation of sustained benefit from 61.5% at 12 months to 29% at 24 months, suggesting that some patients may require repeat or maintenance therapy [29].

#### 4.2.2. Nitroglycerin Use

**Figure 3:**
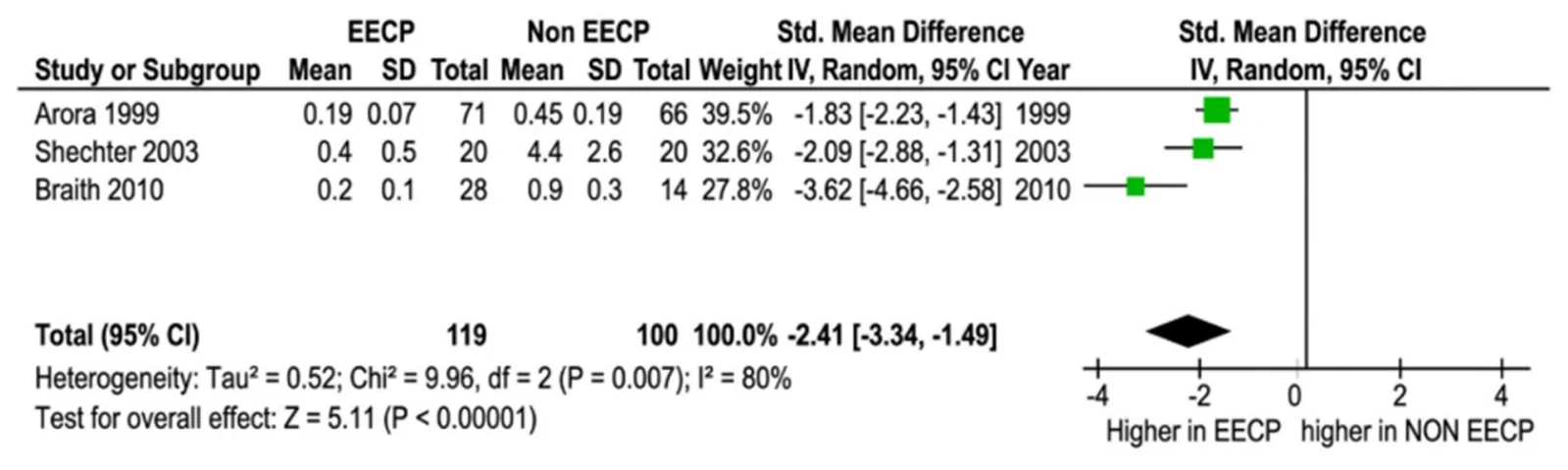
Forest plot of the effect of enhanced external counterpulsation [EECP] on nitroglycerin use compared with control therapy [5,15,16].

Five studies, including Arora, Shechter, Braith, May, and Wu, evaluated daily or weekly nitroglycerin consumption [5,15,16,17,24]. EECP was associated with a marked reduction in nitrate use [SMD −2.41; 95% CI, −3.34 to −1.49; *p* < 0.00001], with high heterogeneity [I^2^ = 80%], as seen in Figure 3. This reduction in rescue nitroglycerin paralleled observed reductions in angina frequency and provides a clinically intuitive measure of symptomatic benefit; its unusually large magnitude, however, reflects a substantial contribution from uncontrolled before–after comparisons and should be interpreted accordingly. Representative findings include a decline in daily angina attacks from 2.7 to 0.9 episodes, reported by May et al. [*p* < 0.005], and a reduction in nitroglycerin use from 4.2 ± 2.7 to 0.4 ± 0.5 tablets per day, reported by Shechter et al. [*p* < 0.001] [5,17]. In the MUST-EECP trial, a greater proportion of patients receiving active therapy reported fewer angina episodes than those receiving sham treatment [*p* < 0.05] [15].

### 4.3. Effects on Exercise Capacity

#### 4.3.1. Six-Minute Walk Test

**Figure 4:**
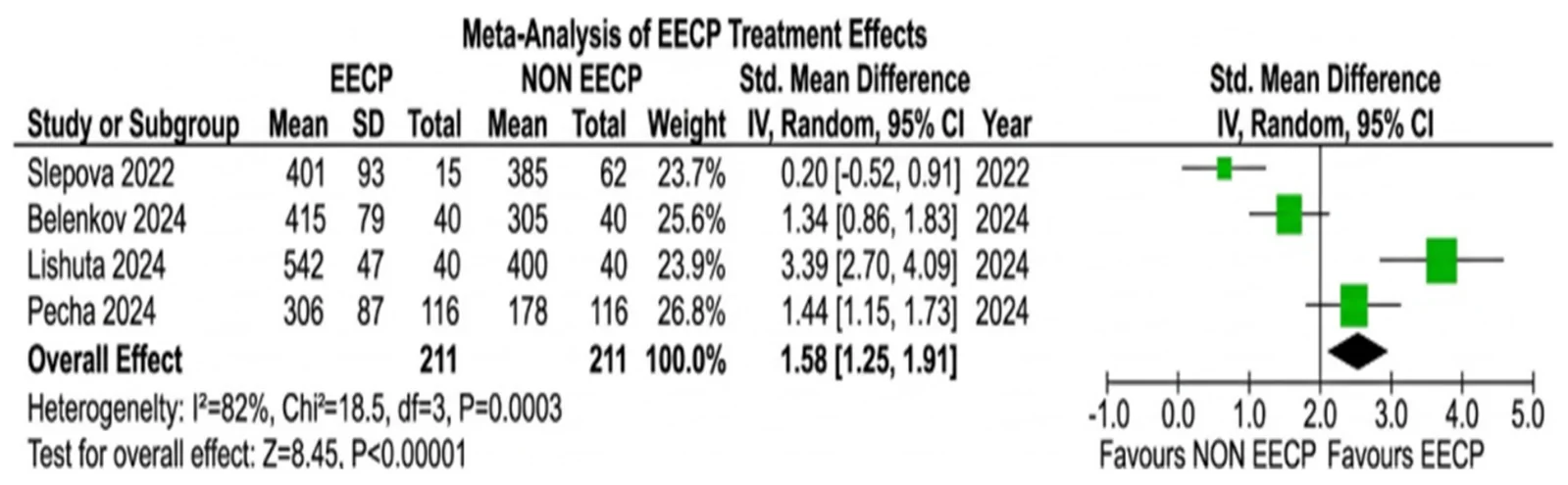
Forest plot of the effect of enhanced external counterpulsation [EECP] on six-minute walk test [6MWT] distance compared with control therapy [7,20,21,22].

Four studies, including Slepova, Belenkov, Lishuta, and Pecha, contributed 422 patients to the pooled analysis of 6MWT performance [7,20,21,22]. EECP significantly improved walking distance [SMD 1.59; 95% CI, 0.64 to 2.54; *p* = 0.001], although heterogeneity was high [I^2^ = 93%], as seen in Figure 4. Absolute increases ranged from approximately 46 to 128 m, suggesting a clinically meaningful improvement in submaximal functional capacity. Pecha et al. reported a mean increase of 128 m, a 72% improvement [*p* < 0.001], with similar gains among men and women [7]. Wu et al. reported a 46 m improvement [*p* < 0.001], and Slepova et al. observed a median increase of 51 m [*p* < 0.05] [24].

The EXCEL study series provides additional evidence that treatment intensity may influence functional response. Compared with a 6.6% increase in 6MWT distance among controls, one annual EECP course was associated with a 24.9% increase, twice-yearly courses with a 44.5% increase, and combined courses with a 58.6% increase. At 12 months, a more than 20% improvement in 6MWT distance was achieved by 97.5% of patients in the twice-yearly EECP group compared with 7.7% in the placebo group [20].

#### 4.3.2. Treadmill Exercise Testing

**Figure 5:**
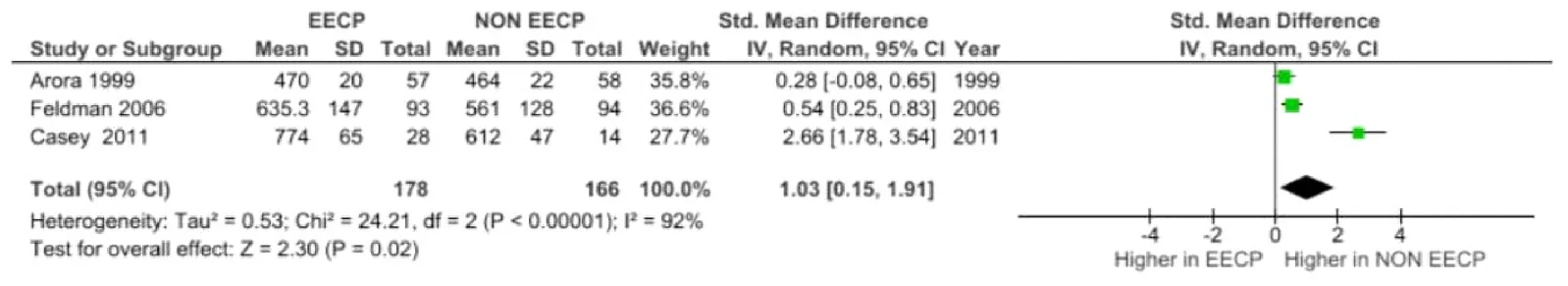
Forest plot of the effect of enhanced external counterpulsation [EECP] on treadmill exercise testing compared with control therapy [6,15,23].

Three studies, including Arora 1999, Feldman 2006, and Casey 2011, evaluated treadmill exercise duration or time to ischemia [6,15,23]. EECP was associated with significant improvement in exercise testing parameters [SMD 1.03; 95% CI, 0.15 to 1.91; *p* = 0.02], again with high heterogeneity [I^2^ = 92%]. These findings suggest that EECP improves not only submaximal walking capacity but also maximal or symptom-limited exercise tolerance under stress-testing conditions, as seen in Figure 5.

### 4.4. Effects on Quality of Life

**Figure 6:**
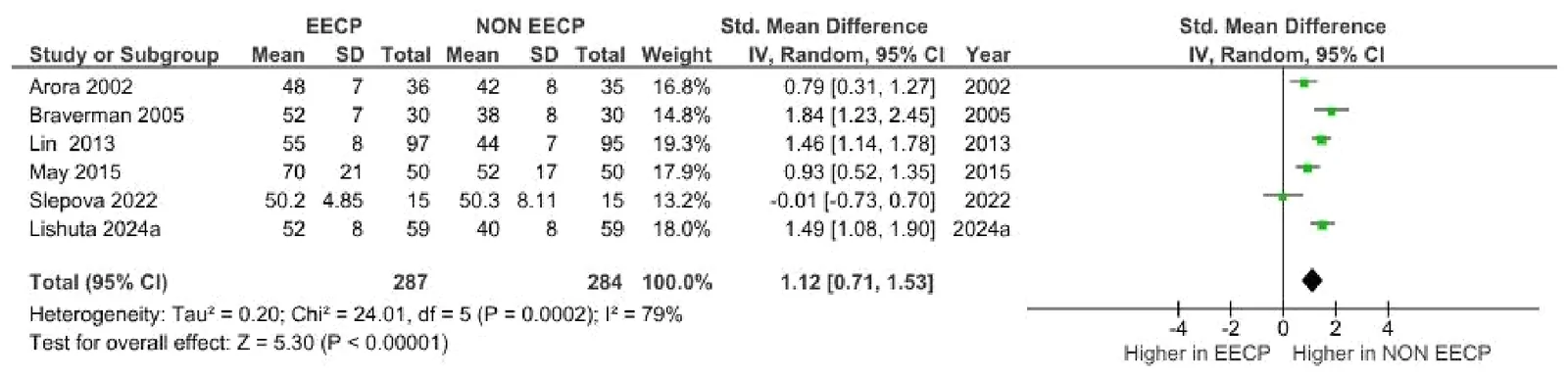
Forest plot of the effect of enhanced external counterpulsation [EECP] on health-related quality of life compared with control therapy [17,21,22,25,26,30].

Six studies, including Arora, Braverman, Lin, May, Slepova, and Lishuta, evaluated quality-of-life outcomes in 571 participants, as seen in Figure 6 [17,21,22,25,26,30]. EECP was associated with significant improvement in quality-of-life scores [SMD 1.12; 95% CI, 0.71 to 1.53; *p* < 0.00001], with substantial heterogeneity [I^2^ = 79%]. Improvements spanned physical, social, emotional, and cardiac-specific domains and were observed across multiple validated instruments. In the MUST-EECP substudy, Arora et al. reported greater improvement among patients receiving active EECP than among those receiving sham therapy across all 9 assessed dimensions, and several domains, including bodily pain, social functioning, and cardiac-specific quality of life, remained improved at 1 year [15].

Braverman et al. reported meaningful functional improvement after treatment with the LIFEware System, with fatigue scores increasing from 41.0 to 59.8, life satisfaction from 45.5 to 68.2, social engagement from 60.4 to 77.9, and role participation from 63.2 to 78.8; further improvement was observed at 6 months, and 83% of patients achieved the prespecified threshold for clinical significance [25]. Wu et al. additionally evaluated cardiac anxiety and reported significant improvement after EECP [*p* < 0.05] [24]. These findings are clinically important because the burden of refractory angina extends beyond ischemic symptoms alone: fear of exertion, recurrent chest pain, and anticipatory anxiety may reinforce physical inactivity and impair recovery.

### 4.5. Safety Profile and Adverse Events

EECP was generally well tolerated across the included studies. Casey et al. reported complete adherence among participants, and Bondesson et al. reported completion of the full EECP treatment course without adverse cardiac events [6,27]. Nevertheless, tolerability is a clinically relevant consideration because active compression can cause musculoskeletal discomfort or skin-related complications.

Adverse events were usually mild and most commonly included leg pain, skin irritation or abrasion at cuff-contact sites, paresthesia, and treatment-related discomfort. In the EXCEL trial series, unexpected treatment-associated effects, including erections, were reported in 7.1% of treated patients [20]. Treatment withdrawal occurred more frequently with active EECP than with sham therapy; McKenna et al. reported a relative risk of withdrawal due to adverse events of 2.13 [95% CI, 1.35 to 3.38], likely reflecting the mechanical intensity of active cuff compression [31].

Serious adverse events were uncommon but warrant consideration given the high baseline cardiovascular risk of the population studied. Erdling et al. reported two deaths from myocardial infarction during treatment and three additional deaths during follow-up in a vulnerable cohort, whereas other studies, including Bondesson et al., reported no cardiovascular adverse events during EECP therapy [27,29]. Commonly cited contraindications include acute coronary syndrome, decompensated heart failure, severe valvular disease, arrhythmias that impair counterpulsation timing, uncontrolled hypertension, deep vein thrombosis, significant peripheral vascular disease, and clinically important bleeding diathesis or coagulopathy.

### 4.6. Sensitivity Analysis Restricted to Randomized Sham-Controlled Trials

Two studies met the criterion of randomized allocation with a sham comparator in the refractory-angina population: MUST-EECP (Arora et al., with its quality-of-life substudy) and the sham-controlled physiologic trial of Braith et al. [15,16,30]. Because these trials informed different endpoints, a separate pooled estimate could not be generated for any single outcome, and the randomized evidence is therefore summarized at the trial level.

For angina class, the randomized signal rests on Braith et al., in which 62% of actively treated patients improved by at least one CCS class versus none in the sham group (*p* < 0.001) [16]. For rescue nitrate use and angina frequency, the randomized signal rests on MUST-EECP, in which active counterpulsation was associated with fewer anginal episodes than sham treatment (*p* < 0.05) and with prolongation of time to exercise-induced ST-segment depression [15]. For quality of life, the MUST-EECP substudy reported greater improvement with active than with sham therapy across all assessed dimensions, with several domains remaining improved at 12 months [15,30].

When restricted to these randomized data, the direction of effect was concordant with the primary pooled analyses, but the magnitude was more modest and the estimates less precise, consistent with the expectation that uncontrolled before–after cohorts inflate apparent effect sizes. The scarcity of sham-controlled trials (only two of 26 included studies) is itself a principal finding and frames the interpretation of every pooled estimate reported above.

### 4.7. Certainty of the Evidence (GRADE)

The certainty of evidence was assessed using the GRADE framework across the domains of risk of bias, inconsistency, indirectness, imprecision, and publication bias. Overall, the certainty of evidence ranged from low to very low across the evaluated outcomes. Evidence for improvement in Canadian Cardiovascular Society (CCS) angina class, nitroglycerin use, and health-related quality of life was rated as low certainty, primarily because most included studies were observational, and substantial statistical heterogeneity was present [I^2^ = 96%, 80%, and 79%, respectively]. The certainty of evidence for six-minute walk distance, treadmill exercise testing, and safety outcomes was rated as very low, owing to serious risk of bias, considerable heterogeneity [I^2^ > 90% for exercise outcomes], inconsistent reporting of adverse events, small sample sizes, and the limited number of randomized sham-controlled trials. Despite these limitations, the available evidence consistently suggests that enhanced external counterpulsation (EECP) improves angina severity, reduces nitroglycerin use, enhances exercise capacity, and improves quality of life in patients with chronic refractory angina. However, confidence in these effect estimates remains limited by the predominance of non-randomized studies and the paucity of high-quality randomized evidence, highlighting the need for additional well-designed sham-controlled randomized clinical trials.

### 4.8. Risk-of-Bias Assessment

Risk of bias was assessed using the Cochrane Risk of Bias 2 (RoB 2) tool for randomized controlled trials and the Risk of Bias in Non-randomized Studies of Interventions (ROBINS-I) tool for observational studies, including prospective cohorts, registries, and observational substudies. Three publications (Cohn 2006, McKenna 2009, and Picichè 2018) were excluded from the risk-of-bias assessment because they were narrative reviews or health technology/economic reports that did not include original empirical patient-level data [4,31,32]. For clarity of presentation, the ROBINS-I assessment is summarized using a streamlined matrix comprising domains D1–D7, representing the study timeline from confounding at baseline through selection, intervention classification, deviations from intended interventions, missing data, outcome measurement, and selective reporting. Risk of bias for each domain is displayed using a traffic-light color scheme: green [low risk], yellow [moderate risk], and red [serious risk].

**Figure 7:**
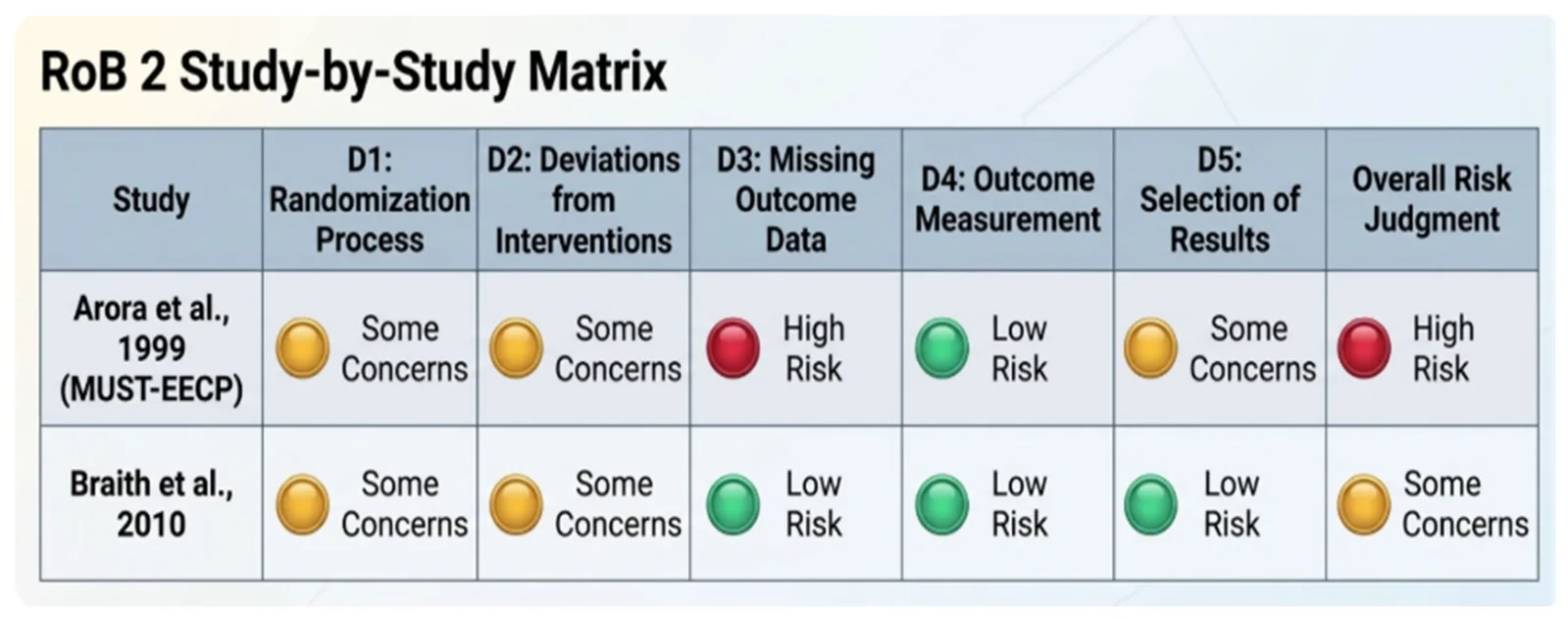
Study-by-study matrix for ROB2 [15,16].

**Figure 8:**
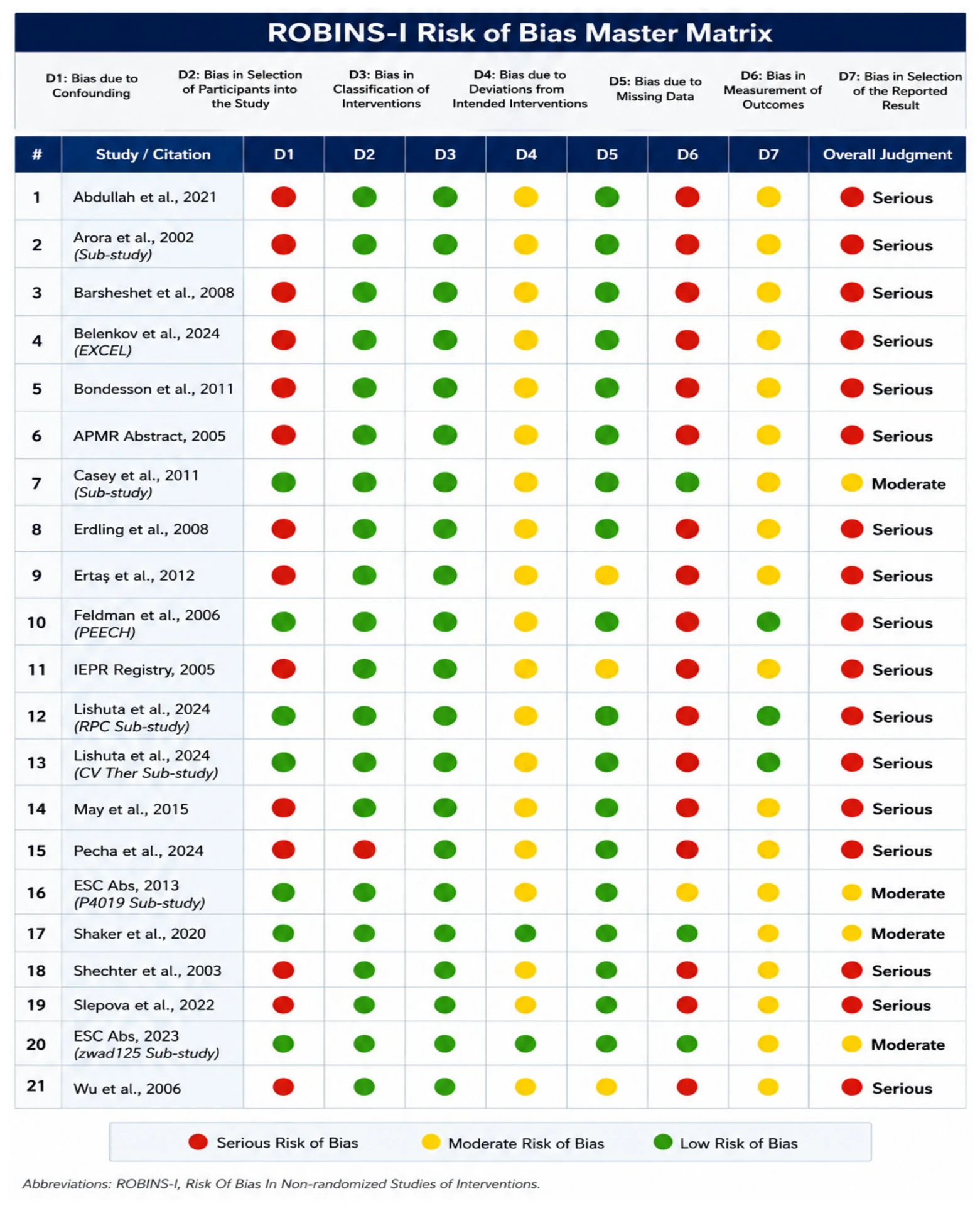
ROBINS-I risk-of-bias assessment of included non-randomized studies [5,6,7,8,9,10,12,17,18,20,22,23,24,25,27,28,29,30,33,34].

Across both risk-of-bias assessment tools, the predominant limitation was measurement bias arising from the absence of sham-device blinding, which particularly affected effort-dependent outcomes, such as the six-minute walk test [6MWT] and subjective symptom-based measures. In contrast, trial substudies demonstrated robust protection against confounding by preserving the original randomized treatment allocation, while studies evaluating objective laboratory biomarkers [e.g., flow cytometry] exhibited the greatest internal validity and measurement reliability, as seen in Figure 7 and Figure 8.

## 5. Discussion

### 5.1. Interpretation of Clinical Efficacy

Effective and durable treatment options remain limited for patients with refractory angina who have exhausted conventional revascularization strategies [32]. The present meta-analysis supports EECP as an effective noninvasive adjunct for this population, consistent with earlier clinical observations that counterpulsation may provide meaningful symptomatic relief when PCI and CABG are no longer feasible [4]. The pooled reductions in CCS class and nitroglycerin use suggest clinically important improvement in day-to-day angina burden, and these findings align with the pivotal MUST-EECP trial, which demonstrated that active counterpulsation prolongs time-to-exercise-induced ST-segment depression and thereby raises the ischemic threshold [15]. Importantly, the concordance of subjective and objective endpoints, rather than the absolute magnitude of any single pooled estimate, is the most persuasive feature of the evidence.

The inclusion of Feldman 2006 strengthens the functional signal by showing improvement across two complementary measures: submaximal performance by 6MWT and maximal or symptom-limited performance by treadmill exercise testing [23]. These effects should not be interpreted primarily as changes in left ventricular systolic function; rather, they likely reflect improved ischemic tolerance, peripheral vascular function, and exercise conditioning. Casey et al. similarly reported improvements in peak oxygen uptake, total exercise duration, and time to angina, supporting a physiologic basis for the observed symptomatic benefit [6].

### 5.2. Mechanisms of Action: The Shear-Stress Hypothesis

The benefits of EECP likely extend beyond transient hemodynamic unloading. Repeated sessions appear to induce vascular conditioning through endothelial shear stress, improved vasodilator signaling, reduced arterial stiffness, and collateral recruitment, providing a plausible bridge between the mechanical intervention and the observed improvements in angina, exercise tolerance, and quality of life.

Endothelial function and shear stress: Chronic refractory angina is frequently associated with endothelial dysfunction. EECP increases diastolic blood flow and pulsatile shear stress along the vascular endothelium, functioning in some respects as a form of passive vascular exercise. This stimulus may enhance nitric oxide bioavailability, improve flow-mediated dilation, reduce arterial stiffness, and decrease myocardial oxygen demand by lowering afterload. Braith et al. demonstrated improvement in peripheral artery flow-mediated dilation after EECP, and related studies have reported favorable changes in vascular function and antithrombogenic activity after treatment [5,8,29,35]. Ryabov et al. reported improved endothelial function and antithrombogenic endothelial activity, whereas Ertaş et al. observed favorable effects on mean platelet volume, suggesting a possible reduction in platelet reactivity [8,28].

Angiogenesis and collateral recruitment: EECP may promote angiogenesis and collateral vessel development by increasing the shear-mediated release of vascular endothelial growth factor [VEGF], nitric oxide, and other angiogenic mediators [11,16]. Ambari et al. reported preservation of VEGF-A and VEGF receptor-2 signaling after external counterpulsation, supporting the biologic plausibility of vascular remodeling and collateral recruitment [11]. Witarsa et al. further demonstrated modulation of angiopoietin pathways, suggesting that EECP may influence multiple angiogenic signaling networks [12]. This “natural bypass” concept may help explain why clinical benefits can persist beyond the active treatment period in selected patients.

Diastolic augmentation: EECP augments coronary perfusion pressure during diastole, when coronary flow is greatest, and deflates before systole to reduce afterload [36]. Invasive hemodynamic studies have confirmed that EECP increases intracoronary pressure and Doppler flow velocity while simultaneously unloading the left ventricle [37,38]. These effects may improve oxygen delivery to ischemic territories, particularly when distal vascular beds are maximally vasodilated and perfusion is pressure dependent.

These mechanisms may be relevant beyond obstructive epicardial coronary disease. In patients with angina and non-obstructive coronary arteries [ANOCA] or INOCA, symptoms are often mediated by coronary microvascular dysfunction rather than flow-limiting stenosis. Recent data suggest that EECP may improve CCS class, 6MWT performance, and Duke Activity Status Index in ANOCA, supporting the hypothesis that endothelial and microvascular conditioning may be clinically meaningful in non-obstructive ischemic syndromes [39].

### 5.3. Durability and Maintenance Regimens

The durability of EECP benefit varies across studies. Registry data from the International EECP Patient Registry suggest that symptomatic improvement may persist for up to 2 years in many patients, although benefit is not uniformly permanent [19,21]. The divergent long-term signals from May et al. and Erdling et al. described above illustrate that clinical response may attenuate over time in a subset of patients [17,29].

This observed “wearing-off” effect has prompted interest in maintenance or repeated EECP protocols. The EXCEL study series suggests a dose–response relationship, with twice-yearly courses associated with greater event-free survival and more durable functional improvement than single annual courses [20,33]. Adjunctive pharmacologic strategies may also modify response: Shaker et al. reported improved outcomes when trimetazidine was combined with EECP, suggesting possible synergy between metabolic modulation and mechanical counterpulsation [40]. Together, these findings support prospective evaluations of individualized maintenance strategies rather than routine reliance on a single 35 h course for all patients.

### 5.4. Predictors of Response: Diabetes and Baseline Disease Severity

Response to EECP is unlikely to be uniform across all patient subgroups. In refractory angina, the relevant determinant may be ischemic burden and microvascular reserve rather than baseline ejection fraction alone. Diabetes mellitus may attenuate response because of endothelial dysfunction, impaired nitric oxide signaling, and microvascular disease. Sahebjami et al. reported a marked reduction in pain frequency among nondiabetic patients [*p* < 0.0005], whereas the change among diabetic patients did not reach statistical significance [41]. These findings suggest that diabetic patients may require individualized treatment strategies; however, prospective trials are needed before longer or intensified EECP courses can be recommended.

Baseline angina severity may also influence response. Erdling et al. reported more durable improvement among patients with CCS class III to IV symptoms than among those with CCS class II symptoms [29]. This observation supports prioritization of EECP for patients with severe, persistent, treatment-limiting symptoms, although it should be interpreted cautiously given the observational nature of the available evidence.

### 5.5. Expanding the Role of EECP in INOCA and MINOCA

Historically, EECP research has focused on refractory angina caused by obstructive epicardial coronary artery disease. Contemporary practice increasingly recognizes INOCA and ANOCA as clinically important syndromes driven by coronary microvascular dysfunction, vasomotor abnormalities, endothelial dysfunction, or mixed mechanisms. Because EECP enhances shear stress and endothelial-derived vasoactive signaling, it may directly target the functional vascular impairment that contributes to symptoms in these populations. Retrospective data from specialized EECP centers involving 101 patients with ANOCA showed that a full 35-session course was associated with at least a 1-class CCS improvement in 70.3% of patients [21].

The role of EECP after MINOCA remains less certain. Precision diagnostic evaluation and etiology-guided pharmacotherapy remain foundational for MINOCA and INOCA management, yet a subset of patients continues to experience persistent angina despite targeted medical therapy. In patients whose symptoms are driven by microvascular ischemia or endothelial dysfunction, EECP is a biologically plausible adjunctive therapy. Dedicated prospective studies are needed to define which non-obstructive ischemic phenotypes are most likely to benefit, the durability of response, and the optimal treatment schedule.

### 5.6. Safety Versus Benefit Profile

The safety profile of EECP is generally favorable, but tolerability should not be minimized. Active compression may cause discomfort, skin irritation, paresthesia, and withdrawal from therapy, as reflected by a higher adverse-event-related discontinuation rate compared with sham treatment [31]. Serious cardiovascular events were uncommon and occurred predominantly in populations with high baseline risk. For appropriately selected patients without contraindications who can tolerate mechanical compression, the balance of benefit and risk appears favorable.

### 5.7. Psychosocial Impact and Quality of Life

For many patients with refractory angina, quality of life is shaped not only by ischemic symptoms but also by fear-avoidance behavior, loss of independence, and anxiety surrounding recurrent chest pain. The improvement in quality-of-life outcomes observed in this meta-analysis likely reflects both reduced symptom burden and renewed confidence with activity. Wu et al. reported improvement in cardiac anxiety after EECP, suggesting that symptom improvement may interrupt the cycle of chest pain, fear, inactivity, and deconditioning [24]. Similar psychosocial benefits have been reported in other cardiovascular populations treated with EECP, including patients with paroxysmal atrial fibrillation [34]. In the MUST-EECP substudy, improvements in psychosocial function and vitality persisted at 12 months, supporting the possibility that EECP can improve both physiologic and patient-centered outcomes [15,22].

### 5.8. Limitations

Several limitations should temper interpretation. First and most importantly, the pooled estimates combine sham-controlled randomized trials with uncontrolled single-arm before–after cohorts and registry analyses. Single-arm pre–post comparisons lack a concurrent control for placebo response, regression to the mean, and co-intervention, and they tend to yield larger apparent effects; the very large summary estimates for nitroglycerin use and CCS class in particular are driven substantially by such designs and are best read as hypothesis-generating rather than as precise treatment effects. A prespecified sensitivity analysis restricted to randomized sham-controlled trials would clarify the magnitude of benefit attributable to EECP itself and is a priority for future work. Second, heterogeneity was substantial across all outcomes (I^2^ ≥ 79%), reflecting differences in baseline severity, comparator, design, follow-up, protocol, and outcome definitions; formal exploration through subgroup analysis or meta-regression was limited by the number and reporting of contributing studies. Third, blinding is intrinsically difficult because active cuff inflation is hard to mask, and low-pressure sham protocols may exert residual hemodynamic effects; subjective endpoints, such as quality of life, are therefore vulnerable to expectation bias, although concordant improvements in 6MWT, treadmill testing, and nitrate use support a genuine physiologic signal. Fourth, higher withdrawal with active therapy may introduce attrition bias. Finally, many included studies were small, non-randomized, or registry based, underscoring the need for larger contemporary sham-controlled trials, prospective registration, and standardized reporting.

## 6. Conclusions

In this systematic review and meta-analysis, EECP was associated with significant improvement in angina severity, nitroglycerin use, functional capacity, and quality of life among patients with chronic refractory angina. Although minor adverse events and higher withdrawal rates occur more frequently with active therapy than with sham treatment, EECP appears safe and, in appropriately selected patients, clinically beneficial. Because the pooled estimates draw on both randomized and non-randomized data and are accompanied by high heterogeneity, they are best regarded as a consistent and clinically plausible signal of benefit rather than a definitive quantification of effect. On balance, the evidence supports EECP as a reasonable adjunctive therapy for patients with persistent, treatment-limiting angina who are not candidates for further revascularization. Future studies should prioritize standardized EECP protocols, maintenance strategies, patient-level predictors of response, formal risk-of-bias and certainty grading, and prospective evaluation in INOCA, ANOCA, and selected post-MINOCA populations.

## Figures and Tables

**Table 1:** Summary of included study designs and clinical contributions.

Study Group/Representative Studies	Design/Population	Primary Contribution to Synthesis
MUST-EECP/Arora et al. [15]	Randomized sham-controlled trial in chronic stable or refractory angina	Exercise-induced ischemia, angina episodes, and quality-of-life outcomes
Shechter et al. [5]	Prospective study of refractory angina	CCS class, nitroglycerin use, and endothelial function
Braith et al. [16]	Randomized sham-controlled physiologic study	CCS class and flow-mediated dilation
May et al. [17]	Prospective cohort with 1-year follow-up	Angina frequency, nitroglycerin use, and durability of benefit
Lawson/IEPR cohorts [18,19]	Large registry-based cohorts	Long-term angina status and real-world durability
Slepova, Belenkov, Lishuta, and Pecha [7,20,21,22]	Prospective contemporary cohorts	6MWT performance, treatment-intensity response, and functional capacity
Casey/Feldman studies [6,23]	Exercise physiology cohorts	Treadmill exercise duration, time to ischemia, and peak oxygen uptake
Wu/Braverman/Lin [24,25,26]	Prospective quality-of-life studies	Patient-reported function, cardiac anxiety, and social and role participation

**Table 2:** Pooled random-effects estimates for EECP versus control.

Outcome	Studies [*n*]	Participants	SMD [95% CI]	*p*	I^2^
CCS angina class	8	1860	−1.56 [−2.54, −0.58]	0.002	96%
Nitroglycerin use	5	219	−2.41 [−3.34, −1.49]	<0.00001	80%
6 min walk test	4	422	1.59 [0.64, 2.54]	0.001	93%
Treadmill exercise testing	3	344	1.03 [0.15, 1.91]	0.02	92%
Quality of life	6	571	1.12 [0.71, 1.53]	<0.00001	79%

SMD, standardized mean difference; CI, confidence interval; CCS, Canadian Cardiovascular Society. Negative SMDs favor EECP for CCS class and nitroglycerin use; positive SMDs favor EECP for 6MWT, treadmill exercise testing, and quality of life. Participant totals are shown where reported by contributing studies.

## Data Availability

No new data were created or analyzed in this study. Data sharing is not applicable to this article.
